# Influence of dietary and physical activity restriction on pediatric adenotonsillectomy postoperative care in Brazil: a randomized clinical trial^☆^

**DOI:** 10.1016/j.bjorl.2017.01.007

**Published:** 2017-02-22

**Authors:** Denise Manica, Leo Sekine, Larissa S. Abreu, Michelle Manzini, Luísi Rabaioli, Marcel M. Valério, Manoela P. Oliveira, João A. Bergamaschi, Luciano A. Fernandes, Gabriel Kuhl, Cláudia Schweiger

**Affiliations:** Hospital de Clínicas de Porto Alegre, Porto Alegre, RS, Brazil

**Keywords:** Adenoidectomy, Motor activity, Diet, Pain, Tonsillectomy, Adenoidectomia, Atividade motora, Dieta, Dor, Tonsilectomia

## Abstract

**Introduction:**

Although culturally food and physical activity restriction are part of the routine postoperative care of many Brazilian surgeons, current evidences from other countries support no such recommendations.

**Objective:**

To determine whether dietary and physical restriction effectively lead to a decrease on postoperative complications of adenotonsillectomy in children when compared to no restriction.

**Methods:**

We have designed a randomized clinical trial comparing two intervention: no specific counseling on diet or activity (Group A), and restriction recommendations on diet and physical activities (Group B). Caregivers completed a questionnaire on observed pain, diet and activity patterns, and medications administered. Parameters were compared at the 3rd and at the 7th postoperative day between intervention groups.

**Results:**

We have enrolled a total of 95 patients, 50 in Group A and 45 in Group B. Fourteen patients were lost to follow up. Eventually, 41 patients in group A and 40 in Group B were available for final analysis. Mean age in months (A = 79.5; SD = 33.9/B = 81.1; SD = 32.6) and sex (A = 58% male; B = 64.4% male) were equivalent between groups. Pain, evaluated through visual analog scale in the 3rd (A = 2.0; IQR 1–6/B = 4.5; IQR 2–6; *p* = 0.18) and in the 7th (A = 1.0; IQR 1.0–4.5/B = 2.0; IQR 1.0–4.7; *p* = 0.29) postoperative days, was not different between groups, as was the amount of analgesics administered. Dietary and physical activity patterns also showed no statistically significant differences between groups.

**Conclusion:**

Dietary and activity restriction after adenotonsillectomy does not seem to affect patients’ recovery. Such information may impact considerably on the social aspects that involve a tonsillectomy, reducing the working days lost by parents and accelerating the return of children to school.

## Resumo

**Introdução:**

Embora culturalmente as restrições dietéticas e de atividade física sejam parte do cuidado pós-operatório de rotina de muitos cirurgiões brasileiros, evidências atuais de outros países não apoiam tais recomendações.

**Objetivo:**

Determinar se as restrições dietéticas e físicas efetivamente levam a uma diminuição das complicações pós-operatórias da adenotonsilectomia em crianças quando comparadas com cuidados sem nenhuma restrição.

**Método:**

Realizamos um ensaio clínico randomizado comparando duas intervenções: nenhum aconselhamento específico sobre dieta ou atividade física (Grupo A) e recomendações de restrições dietéticas e de atividades físicas (Grupo B). Os cuidadores preencheram um questionário sobre a dor, a dieta e os padrões de atividade observados, e os medicamentos administrados. Os parâmetros foram comparados no 3° e no 7° dia do pós-operatório entre os grupos de intervenção.

**Resultados:**

Avaliamos um total de 95 pacientes, 50 no Grupo A e 45 no Grupo B. Quatorze pacientes foram perdidos no seguimento. Subsequentemente, 41 pacientes do grupo A e 40 do grupo B estavam disponíveis para a análise final. A média de idade em meses (A = 79,5, DP = 33,9/B = 81,1, DP = 32,6) e sexo (A = 58% do sexo masculino, B = 64,4% do sexo masculino) foram equivalentes entre os grupos. A dor, avaliada através da escala visual analógica no 3° (A = 2,0; IIQ: 1-6/B = 4,5; IIR 2-6; *p* = 0,18) e no 7° (A = 1,0; IIQ 1,0-4,5/B = 2,0; IIQR 1,0-4,7; *p* = 0,29) dia do pós-operatório, não foi diferente entre os grupos, assim como a quantidade de analgésicos administrados. Os padrões dietéticos e de atividade física também não mostraram diferenças estatisticamente significantes entre os grupos.

**Conclusão:**

A restrição dietética e de atividade física após a adenotonsilectomia não parece afetar a recuperação dos pacientes. Tal informação pode ter um impacto considerável nos aspectos sociais que envolvem uma tonsilectomia, reduzindo os dias de trabalho perdidos pelos pais e acelerando o retorno das crianças à escola.

## Introduction

Adenotonsillectomy is one of the most common surgical procedures performed in the United States. Although the exact number of such procedures currently performed in Brazil is not known, they are part of the routine of most otolaryngologists.

Adenotonsillectomy is a potential curative surgical procedure for patients presenting recurrent throat infections and Sleep-Disordered Breathing (SDB) which can both substantially affect child's health status and Quality of Life (QoL).^1^ The benefit of adenotonsillectomy in child's QoL is well documented. Tonsillectomy may improve QoL by reducing throat infections, health care provider visits, and the need for antibiotic therapy. Similarly, it improves sleep disturbance, vocal quality and cognitive and behavioral impairment in children.^2^ Offsetting the benefits of tonsillectomy, surgery complications may include throat pain, postoperative nausea and vomiting, delayed feeding, voice changes, hemorrhage, and rarely death.^2^

Although it is a traditional surgery, the postoperative recovery period still raises doubts and concerns for patient's caregivers, and is culturally seen as restrictive and temporarily disabling. Socioeconomic impacts related to post adenotonsillectomy recovery are considerable, since it is estimated to last between seven and ten days, resulting in school and work absenteeism. There is great heterogeneity among surgeons on the subject of postoperative recommendations, but studies conducted in US^3^, ^4^ and Europe^5^ showed no change in the postoperative outcome of patients advised to restrict the diet and physical activity compared to those with no restrictions. These studies demonstrate that there are no clear benefits in limiting diet during the first 7 days after adenotonsillectomy, with no difference between pain perception, daily dose of analgesics or post surgical complications. There is even evidence for lower pain on non-restrictive group.^5^

## Objective

The aim of this study is to compare post-adenotonsillectomy outcomes between children allocated to postoperative diet and physical activity restrictions, and those without any specific restriction.

## Methods

This randomized trial was approved by the Institutional Research Ethics Committee, under the project number 15-0500. Data were collected between April 2014 and February 2016. Children 3–12 years old admitted for outpatient adenotonsillectomy were invited to participate. Neurologically compromised and syndromic patients were not eligible. Informed consent was obtained verbally and in written form from parents/caregivers. Children were allocated into two groups, no dietary and physical restrictions (Group A), and dietary and physical restrictions (Group B), by a simple randomization method. Children on Group A were advised to resume their usual diet and physical activity according to their tolerance, and patients in Group B were oriented to maintain preferably only liquid and soft foods and to refrain from engaging in physical activities during the first week after surgery. Patients and caregivers allocated to Group B also received a formal medical certificate suspending school and labor activities for 7–10 days, until the follow up visit could be arranged.

Sample size was calculated based on data from the study of Klemetti et al.^6^ and considering a difference between intervention and control group of at least 1.3 points in pain visual analog scale (VAS). Assuming a power of 80% and a significance level of 0.05, sample size estimation resulted in 39 individuals per group.

Surgery was performed using sharp dissection by a resident surgeon in the presence of a supervisor. The same anesthesia protocol was used in all the patients by the same anesthesiologist.

Quantitative variables were compared using Student's *t*-test or Mann–Whitney *U* test, according to the data distribution. Normality of distribution was ascertained with Shapiro–Wilk test. Categorical variables were compared using Pearson Chi-squared test. A significance level of 0.05 was used for all comparisons. Statistical analysis was performed in IBM SPSS Statistics for Windows, Version 20.0 (Armonk, NY: IBM Corp).

Characteristics such as gender, age and race were collected. The date of surgery and its indication, comorbidities as well as the presence of passive smoking were also registered. Parents were asked to answer the questions of the OSA-18 survey.^7^

Patients were also evaluated for mouth breathing facies, degree of palatine tonsil hypertrophy^8^ and bite characteristics (open, cross or normal).

During the surgery, neither was local anesthesia performed, nor routine antibiotics administered (or even in postoperative period).

Telephone interviews were conducted by one of the investigators in the third and seventh postoperative day. Questions answered by the caregiver were: level of pain at the contact day (1–10, graduation based on the Wong-Baker FACES Pain Rating Scale),^9^ pain classification (in continuous, intermittent, rare or absent), doses and name of analgesics used (paracetamol, ibuprofen or dipyrone) and if any other medication was used. Caregivers were not oriented to administer fixed analgesics postoperatively, but instead administer medication on demand based on patients’ symptoms.

Caregivers were also asked to answer if the child diet intake was normal, near normal, moderate or poor. Regarding the return to physical activities, they were asked whether the patient could be classified as bedridden, lethargic but walking, easy fatigability but active, or normal.

## Results

We have followed up 50 individuals in Group A and 45 in Group B. Baseline characteristics of these patients are shown in Table 1. A total of 9 children in Group A and 5 in Group B were lost to follow up due to difficulties in phone contact and/or refusal to answer one or more study questions. Characteristics from these patients were compared between groups, and were considered not to implicate in potential selection bias.

**Table 1:** Baseline characteristics from study groups.

Characteristic	Group A (*n* = 50)^a^	Group B (*n* = 45)^a^
Male	29 (58%)	29 (64.4%)
Age (months)	79.5 (±33.9)	81.1 (±32.6)
Weight (kg)	23 (18.0–30.1)	24 (18.8–31.5)
Ethnicity	Caucasian: 37 (74%)	Caucasian: 32 (71.1%)
	African descent: 5 (10%)	African descent: 4 (8.9%)
	Other: 8 (16%)	Other: 9 (20%)
Surgery indication	Airway obstruction: 40 (80%)	Airway obstruction: 39 (86.7%)
	Recurrent tonsillitis: 1 (2%)	Recurrent tonsillitis: 0 (0%)
	Both: 9 (18%)	Both: 6 (13.3%)
OSA-18	81.7 (20.9)	75.3 (24.9)
Tympanostomy tube	10 (20%)	9 (20%)

a Data expressed as mean (SD), median (interquartile range) or *n* (%).

Final analyses were carried out in 81 patients (Group A = 41 and Group B = 40). Essentially, we found no statistically significant differences in the third (D3) and seventh (D7) postoperative day between groups, concerning visual analog scale (VAS) score for pain, type of pain, amount of physical activity, and feeding pattern. A summary of findings is described in Table 2. Use of analgesics (namely dypirone, acetaminophen and/or ibuprofen) in the last 24 h was assessed on the third and seventh postoperative day. No significant differences were found between Groups A and B regarding the amount of administered doses of these medications (*p* > 0.05).

**Table 2:** Findings summary.

	Group A^a^	Group B^a^	*p*-value
VAS score D3	2 (1–6)	4.5 (2–6)	0.18
VAS score D7	1 (1–4.5)	2 (1–4.7)	0.29
Type of pain D3	Continuous: 5 (12.2%)	Continuous: 7 (17.5%)	0.46
	Intermittent: 14 (34.1%)	Intermittent: 17 (42.5%)	
	Rare: 13 (31.7%)	Rare: 12 (30%)	
	Absent: 9 (22%)	Absent: 4 (10%)	
Type of pain D7	Continuous: 2 (4.9%)	Continuous: 3 (7.5%)	0.45
	Intermittent: 5 (12.2%)	Intermittent: 10 (25%)	
	Rare: 16 (39%)	Rare: 13 (32.5%)	
	Absent: 18 (43.9%)	Absent: 14 (35%)	
Physical activity D3	Bedridden: 0 (0%)	Bedridden: 4 (10%)	0.22
	Lethargic but walking: 10 (24.4%)	Lethargic but walking: 9 (22.5%)	
	Easy fatigability but active: 10 (24.4%)	Easy fatigability but active: 8 (20%)	
	Normal: 21 (51.2%)	Normal: 19 (47.5%)	
Physical activity D7	Bedridden: 1 (2.4%)	Bedridden: 0 (0%)	0.63
	Lethargic but walking: 3 (7.3%)	Lethargic but walking: 3 (7.5%)	
	Easy fatigability but active: 6 (14.6%)	Easy fatigability but active: 9 (22.5%)	
	Normal: 31 (75.6%)	Normal: 28 (70%)	
Feeding pattern D3	Poor: 3 (7.3%)	Poor: 6 (15%)	0.18
	Little with stimulus: 16 (39%)	Little with stimulus: 18 (45%)	
	Almost normal: 12 (29.3%)	Almost normal: 13 (32.5%)	
	Normal: 10 (24.4%)	Normal: 3 (7.5%)	
Feeding pattern D7	Poor: 2 (4.9%)	Poor: 2 (5%)	0.17
	Little with stimulus: 6 (14.6%)	Little with stimulus: 8 (20%)	
	Almost normal: 12 (29.3%)	Almost normal: 19 (47.5%)	
	Normal: 21 (51.2%)	Normal: 11 (27.5%)	

a Data expressed as median (interquartile range) or *n* (%).

## Discussion

This study was initially proposed to assess the actual need for post-adenotonsillectomy restrictions recommended by most otolaryngologists in our country, culturally deemed necessary by surgeons and patients themselves.

A study showing no difference on the recovery rate between restrictive or non-restrictive recommendations was published in 1993,^4^ and another study showing even better postoperative results with non-restrictive guidance^5^ came further. At our institution, however, parents historically used to receive a handout list with diet instructions with a soft diet and limited home activity until the first postoperative medical evaluation, which usually occurs in the first 7–10 days. There was a belief that hard consistency meals could cause pain, nausea and postoperative bleeding from the friable mucosa of the surgical site as well as a delay in normal healing of the pharyngeal mucosa, while excessive physical effort could lead to a circumstantial rise in blood pressure favoring bleeding episodes. So, we found it necessary to reproduce a study in a Brazilian hospital similar to those conducted in institutions abroad, in order to discuss the impact of restrictive habits in our local population of children undergoing adenotonsillectomy.

It was believed initially that we would face a certain resistance from caregivers and physicians in complying with the study. However, surprisingly, we have witnessed a good acceptance from all participants. We would attribute this fact to the potential socioeconomic implications derived from recommending children to refrain from school activities for about a week after tonsillectomy, leading to a significant burden on patient and the family routine activities.

This study demonstrates no clear benefits on limiting diet or activity during the first seven postoperative days after tonsillectomy. No significant differences were found between groups regarding feeding pattern, VAS score, type of pain and return to physical activities after surgery.

In both groups there was a slight predominance of male patients (58% in Group A and 64.4% in Group B), also reported by Mitchell et al.^10^

In this study we have observed no bleeding complications that required intervention and no other ominous outcome. Further studies with longer follow up would be important to demonstrate if complications, such as bleeding, could develop differently in the late postoperative period.

In recent years the number of tonsillectomy indications for SDB has increased.^2^ Tonsillectomy for recurrent throat infections is still acceptable but is restricted to selected cases, as observed in our sample. The observed OSA-18 score was considered similarly high in both groups (Group A = 81.7; Group B = 75.3), which reinforces SDB surgical indication. Also, this finding is in accordance with a similar Brazilian study in the preoperative evaluation of children warranted tonsillectomy, where mean OSA-18 score was 82.8.^11^

A possible limitation of this study is the fact that we have not managed to ensure accuracy of endpoints on follow up visits by applying other methods of pain/diet evaluation. The use of a validated tool like dietary recall or food frequency questionnaire to aid the evaluation of the effective diet followed in both groups could help diminish bias in potential deviations from recommended guidance. Also, as outlined above, our sample was mainly constituted by SDB patients, who are known to present less frequent postoperative complications and pain. Therefore, results should be carefully interpreted when concerning other indications for tonsillectomy. Finally, for the unrestricted diet/activity group, we did not register the number of days patients/caregivers had to skip school/work, so we could not evaluate differences regarding this endpoint either.

## Conclusion

Although culturally involved in the routine of many Brazilian surgeons, food and physical activity restriction is no longer recommended in several developed countries. We have found that such recommendations do not influence the intensity or pattern of pain, or physical activity resumption during the postoperative period. Such information may impact considerably the social aspects that involve a tonsillectomy, reducing workdays lost by parents and anticipating the return of children to school.

## Conflicts of interest

The authors declare no conflicts of interest.
